# An Antagonist Antibody That Inhibits Cancer Cell Growth In Vitro through RACK1

**DOI:** 10.3390/ph17101303

**Published:** 2024-09-30

**Authors:** Ji Hoe Kim, Eun Ji Lee, Kyung Ho Han

**Affiliations:** Department of Biological Sciences and Biotechnology, Hannam University, Daejeon 34054, Republic of Korea

**Keywords:** antibody, cell cycle, RACK1, antibody library, cancer

## Abstract

Background/Objectives: Our research introduces a novel screening method to identify antibodies that can suppress cell proliferation and induce apoptosis. Methods: By using an autocrine signaling system with lentivirus, we developed an antibody screening method based on FACS sorting assays and cell cycle analysis to inhibit tumor growth in vitro. This approach is particularly well suited for studying tumor suppressors. Inducing the G0 phase in tumor cells with specific antibodies may arrest their growth permanently or trigger apoptosis. The cell cycle is composed of tightly regulated phases for cell growth and division, with tumorigenesis or apoptosis occurring when these regulatory mechanisms fail. Results: In our study, we identified RACK1 as a key regulator of cancer cell growth. The H9 antibody against RACK1 selected from a human antibody library effectively suppressed cell proliferation by inhibiting RACK1 function. Conclusions: These findings suggest that RACK1 plays a crucial role in tumor cell cycling and could represent a novel therapeutic target for cancer treatment. Although RACK1 is recognized as a significant target protein in various tumors, no commercial therapeutic agents currently exist. Our results suggest that the H9 antibody could be a promising candidate for the development of novel cancer therapies.

## 1. Introduction

Cancer is the leading cause of death worldwide. In 2022, 20 million new cancer cases and approximately 9.7 million cancer-related deaths were reported globally [1]. According to Statistics Korea, approximately 273,076 cancer cases and 81,818 cancer-related deaths were reported in Korea in 2023. Among these, lung cancer accounts for the highest number of deaths with 18,673 as of 2020, followed by liver cancer, colon cancer, stomach cancer, and breast cancer [2,3].

Cancer is categorized into two main types: solid cancers and hematologic cancers [4]. Hematologic tumors such as leukemia, lymphoma, and myeloma are being treated with various therapies, including tyrosine kinase inhibitors, monoclonal antibodies, checkpoint inhibitors, and CAR-T therapy. While medical advancements have significantly improved treatments for hematologic cancers, progress in treating solid tumors remains slower. Several strategies have been developed for solid cancers, including surgery, chemotherapy, radiotherapy, combinational therapy, targeted therapy, and immunotherapy. For over a century, surgery, chemotherapy, and radiotherapy—used individually, in combination, or in sequence—have been the primary cancer treatment methods. However, these approaches are not always effective in all cases [5].

As part of a previous research group, we recently published antibody screening methods to select antibodies that differentiate or transdifferentiate stem cells into alternative phenotypes. The success of this method relies on the robustness of the selection system. Once a functional antibody is identified, it can be used to identify its target, providing insights into the pathway involved in phenotype generation. We demonstrated notable examples, including the cell migration of different tissues and the formation of a unique cellular morphology [6,7,8,9,10,11,12].

Based on an autocrine signaling system using lentivirus, we developed antibody screening methods utilizing a FACS sorting assay and cell cycle analysis to inhibit tumor growth in vitro. We proposed that this method is ideal for studying tumor suppressors. Using antibodies to induce the G0 phase in cancer cells may halt their growth permanently or trigger apoptosis.

The cell cycle is a tightly regulated series of phases that a cell undergoes to grow and divide, consisting of the G1, S, G2, and M phases, during which cells grow, replicate DNA, prepare for mitosis, and finally divide. Proper progression through the cell cycle is controlled by checkpoints that ensure each phase is completed accurately, preventing DNA damage and ensuring genomic stability. Tumor growth occurs when these regulatory mechanisms fail, leading to uncontrolled cell division and proliferation. Mutations in genes that regulate the cell cycle, such as tumor suppressors and oncogenes, can bypass these checkpoints, resulting in the formation and progression of cancerous tumors. The G0 phase is a resting or quiescent stage in the cell cycle in which cells exit the active cycle and enter a state of dormancy. Cells in the G0 phase are not actively preparing to divide, as they have left the cycle and entered a state of dormancy [13,14,15,16,17]. This study highlights the effectiveness of an antibody targeting annexin-A1 (ANXA1) in inhibiting cancer cell proliferation and tumor growth by arresting the cell cycle in the G1 phase. These findings suggest that therapies focused on arresting the G0 and G1 phases of the cell cycle could offer a promising approach for cancer treatment [18].

In this study, we discovered that the receptor for activated C kinase 1 (RACK1) regulates cancer cell growth. RACK1 has gained attention as a promising target for cancer therapy due to its involvement in many key cellular functions. As a scaffold protein, RACK1 links different signaling pathways that control processes like cell growth, migration, and survival, which cancer often manipulates to promote its progression. In cancers like colorectal, breast, and lung cancer, RACK1 is frequently overexpressed, which is linked to more aggressive tumors and worse outcomes for patients. RACK1 stands out as an intriguing target in cancer research due to its complex, context-dependent role. In some cancers, it drives tumor growth, while in others, such as gastric and pancreatic cancers, it can actually function as a tumor suppressor. This duality makes RACK1 a powerful key to unlocking more effective cancer treatments [19,20,21,22,23,24,25,26].

The H9 antibody against RACK1 was screened from a human antibody library, and the H9 antibody was effective in inducing cell proliferation arrest by blocking RACK1. These findings suggest that RACK1 is involved in the cell cycle of solid cancers and may serve as a novel target for solid cancer treatment.

## 2. Results

### 2.1. Selection System for Inducing G0/G1 Tumor Cell Cycle Arrest

**Figure 1:**
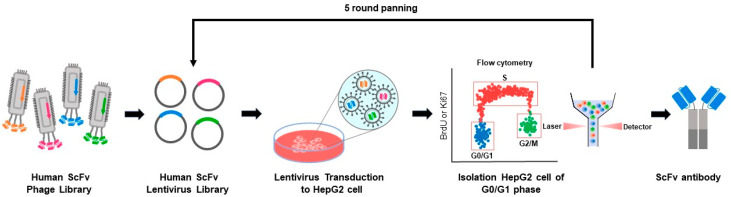
Antibody selection for inducing tumor growth inhibition by in the G0/G1 phase. Scheme for selecting antibodies that inhibit cell growth. Genes from a human ScFv phage library containing 10^8^ members were cloned into a lentiviral vector to produce a lentiviral antibody library. In this library, antibody molecules are anchored to the plasma membrane and displayed on the cell surface. Tumor cells (HepG2) were infected with the antibody library in vitro and cultured for three days. This system functions on an autocrine basis, as each cell expresses a unique antibody. Cells were then sorted based on negative BrdU staining, collected, and analyzed using PCR to identify antibody genes that restrict tumor cell growth. This selection process was repeated for four rounds, from the focused library creation to cell sorting.

We developed a novel antibody screening method to select antibodies that induce tumor growth inhibition (Figure 1). It uses a human single-chain fragment variable (ScFv) phage library to construct a human ScFv lentiviral intracellular combinatorial antibody library containing 10^8^ unique binding clones. The antibodies were displayed on the cell surface using our previously published methods [6,7,8,9,10]. We first infected HepG2 liver cancer cells with the ScFv lentiviral library in vitro. After three days of culture, the cells were selected by flow cytometry. In the first round, cells were sorted by selecting the Ki67-negative population. Afterward, genomic DNA was extracted from these cells, amplified, and used to clone a targeted lentiviral library for the next round of experiments. In the subsequent 2–5 rounds, cells were sorted based on the BrdU-negative population, which indicates the G0/G1 phase of the cell cycle. Each round of cell panning produced a more focused antibody library that is specific to the G0/G1 phase in the HepG2 cell line compared to earlier rounds. With each round, there was an increased population of cells in the G0/G1 phase compared to the control (Figure S1). These results confirm that our new screening method is effective in finding antibodies that induce specific cell cycle phases in cancer cells. To determine if a single antibody from the sorted cell population could suppress cell growth, the sorted cells were collected, and the integrated antibody gene was recovered by PCR. The H9 gene sequence was consistently observed in cells in the G0/G1 phase, leading to the selection of this antibody for further investigation.

### 2.2. Selected H9 Antibody Inhibits Proliferation and Migration of Four Cancer Cell Lines

**Figure 2:**
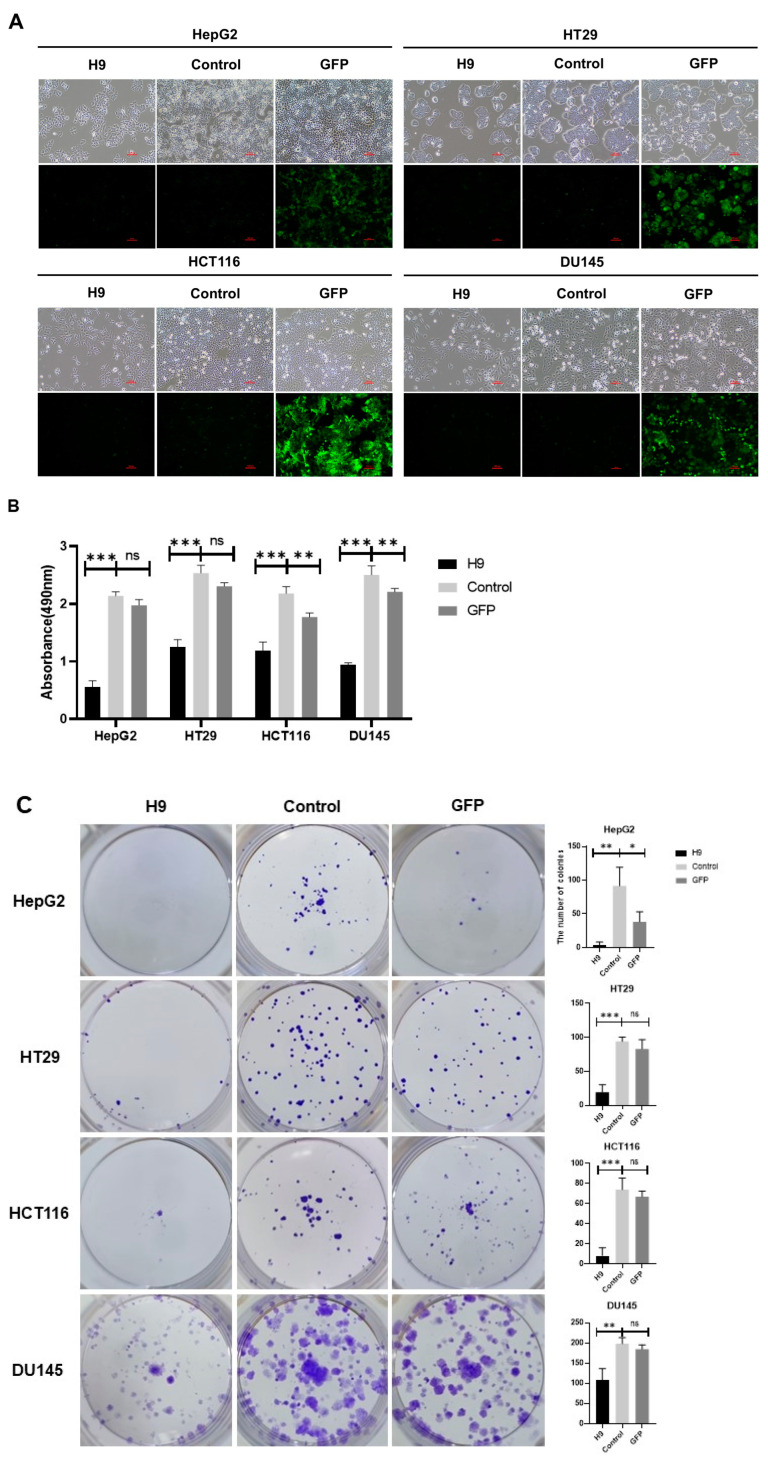

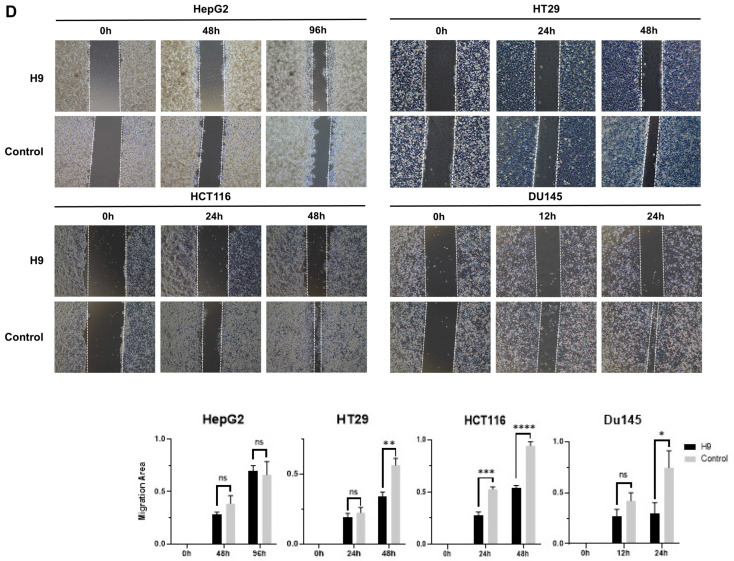
H9 antagonist antibody suppresses tumor cell growth. (**A**) After H9 treatments, HepG2, HT29, HCT116, and DU145 cells in culture. Magnification, 100×. (**B**) Proliferation of HepG2, HT29, HCT116, and DU145 cells treated with H9, as evaluated by the MTS assay. (**C**) Colony formation analysis of HepG2, HT29, HCT116, and DU145 cells, with the number of colonies quantified using ImageJ. (**D**) Wound healing assays showing the effect of H9 on the migration of HepG2, HT29, HCT116, and DU145 cells. Representative images depict scratched and healing wounded areas (marked by white dotted lines) on confluent layers of solid cancer cells transduced with H9. Magnification, 40×. Data are presented as mean ± s.e.m. from an experiment independently repeated at least three times. Statistical analysis was conducted using multiple *t*-tests and one-way ANOVA: ns, not significant; * *p* < 0.05, ** *p* < 0.01, *** *p* < 0.001, **** *p* < 0.0001.

Our study aimed to demonstrate whether selected antibodies from our screening method could induce cancer cell cycle arrest in the G0/G1 phase and trigger cancer cell death. To evaluate the tumor therapeutic ability of the H9 antibody, the H9 clone was inserted into a lentivirus vector. We observed that H9 was effective not only in the HepG2 liver cancer cell line, which was used in our novel screening method, but also in two colon cancer cell lines, HT9 and HCT116, and a DU145 prostate cancer cell line (Figure 2A). We treated these cell lines with H9 for 96 h and investigated cell viability using the MTS assay. The H9-treated cells exhibited a significant reduction in cell growth compared to untreated and GFP-treated control groups (Figure 2B). Furthermore, colony formation assays demonstrated a decrease in the number of colonies formed in tumor cells treated with H9 compared to controls (Figure 2C). Lastly, wound healing assays showed a significant reduction in tumor cell migration, except in HepG2 cells, compared to control groups (Figure 2D). These findings suggest that the H9 antibody markedly reduces the growth, proliferation, and migration of four different cancer cell lines. Importantly, these studies demonstrated that the antibody selected using our novel method induces growth inhibition in cancer cells, not only in the tumor cell line used for screening but also in other tumor cell lines.

### 2.3. Selected H9 Antibody Induces G0/G1 Phase Cell Cycle Arrest

**Figure 3:**
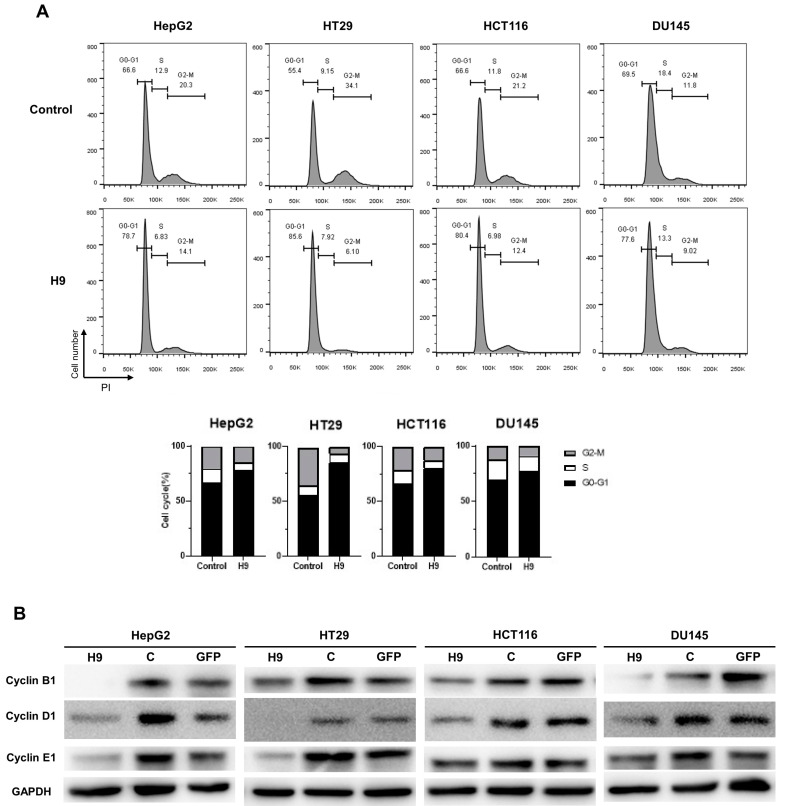
H9 antibody induces G0/G1 phase arrest in tumor cells. (**A**) Cell cycle analysis using flow cytometry following propidium iodide (PI) staining. HepG2, HT29, HCT116, and DU145 cells were treated with the H9 antibody for two days to assess H9-induced cell cycle arrest. (**B**) Western blot analysis of cell cycle regulators in HepG2, HT29, HCT116, and DU145 cells transduced with H9, GFP, control. Cell lysates were labeled with antibodies against cyclin B1, cyclin D1, cyclin E1, and GAPDH.

Because the H9 antibody was derived from a specific G0/G1 phase population in the cell cycle, we next investigated whether H9 specifically leads to the phase of cell cycle arrest. To assess whether H9 regulates cancer cell growth, we used flow cytometry to observe propidium iodide (PI) staining on HepG2, HT29, HCT116, and DU145 cell lines following treatment with H9. After H9 treatment for two days, the proportion of H9-treated tumor cells in the S, G2, and M phases was reduced compared to the controls. Specifically, the percentage of cells in the sub G2/M phase in HepG2, HT29, HCT116, and DU145 cell lines decreased by 6.2%, 28%, 8.8%, and 2.7%, respectively (Figure 3A). Concurrently, the percentage of cells in the sub G0/G1 phase increased by 12.1%, 30.2%, 13.8%, and 8.1%, respectively. Overall, our analysis highlighted a significant reduction in the G2/M peak and an increase in the G0/G1 peak compared to the control (Figure 3A). Cyclin D1 is crucial for regulating the transition from the G1 phase to the S phase in eukaryotic cells, with low protein levels during the G0 phase. Cyclin E1 is also essential for the G1/S transition, and its protein levels increase during the late G1 and S phases. Cyclin B1 reaches high levels during the G2/M phase. To further explore these key protein levels, HepG2, HT29, HCT116, and DU145 cells were treated with H9, and their cell lysates were evaluated using Western blotting with antibodies against cyclin B1, cyclin D1, and cyclin E1. The results confirmed that a significant downregulation of cyclin B1, cyclin D1, and cyclin E1 in four tumor cell lines were stimulated with H9. These results were consistent with the level of cyclins during cell cycle arrest (Figure 3B). Thus, these findings confirmed that H9 induces G0/G1 cell cycle arrest in HepG2, HT29, HCT116, and DU145 cells.

### 2.4. Selected H9 Antibody Induces Apoptosis

**Figure 4:**
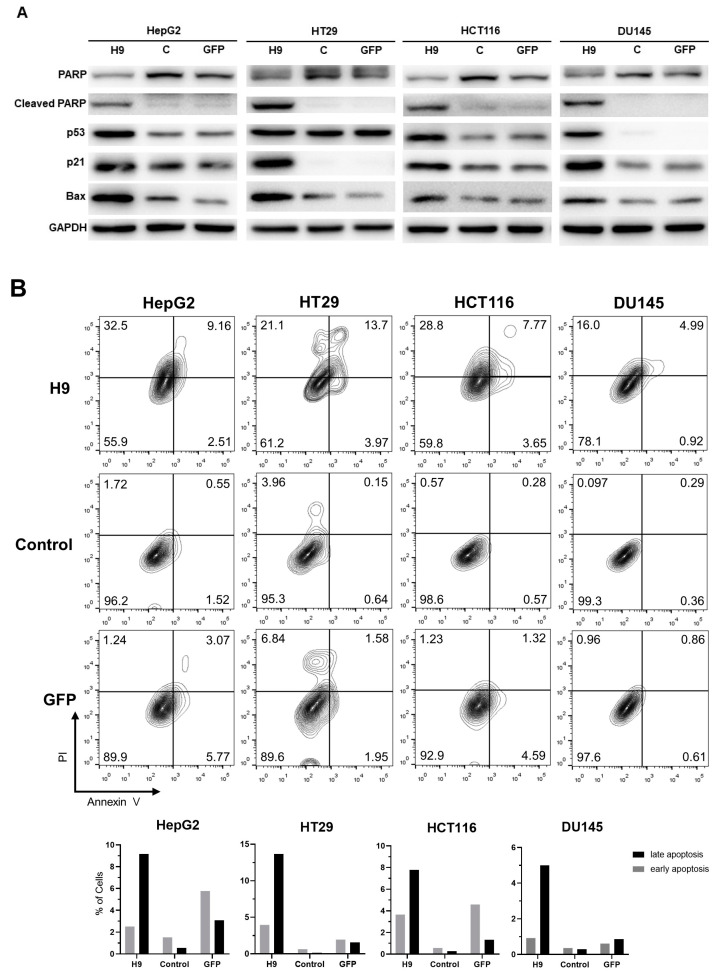
H9 regulates apoptosis in cancer cells. (**A**) Western blot analysis of apoptotic protein expression levels in HepG2, HT29, HCT116, and DU145cells transduced with H9, GFP or control. Cell lysates were labeled with antibodies against PARP, cleaved PARP, p53, p21^Waf1/Cip1^, Bax, and GAPDH. (**B**) Flow cytometry analysis using annexin V/PI staining to detect apoptosis in HepG2, HT29, HCT116, and DU145 cells treated with H9, GFP, and control.

To determine whether H9 induces apoptosis by initiating G0/G1 cell cycle arrest, we investigated the levels of apoptotic cell death markers such as PARP, cleaved PARP, p53, p21^Waf1/Cip1^, and Bax (Figure 4A). Remarkably, the expression levels of cleaved PARP, p53, and p21^Waf1/Cip1^ and Bax, which are apoptotic cell death proteins, were significantly increased following a treatment with the H9 antibody in HepG2, HT29, HCT116, and DU145 cells. Next, we examined the cell death mechanism with PI/annexin V staining through flow cytometry. The results showed that the apoptosis rate (annexin-V+/PI+ or −) was notably higher in H9-treated tumor cell lines compared to the controls. H9 treatment caused an increase in early apoptotic cells by 0.99%, 3.33%, 3.08%, and 0.56% in HepG2, HT29, HCT116, and DU145 cells, respectively, while late apoptotic cells increased by 8.61%, 13.55%, 7.49%, and 4.7% in the same cell lines, respectively (Figure 4B). Taken together, these findings suggest that H9 is involved in apoptosis pathway in tumor cell lines.

### 2.5. Identification of a Target Protein

**Figure 5:**
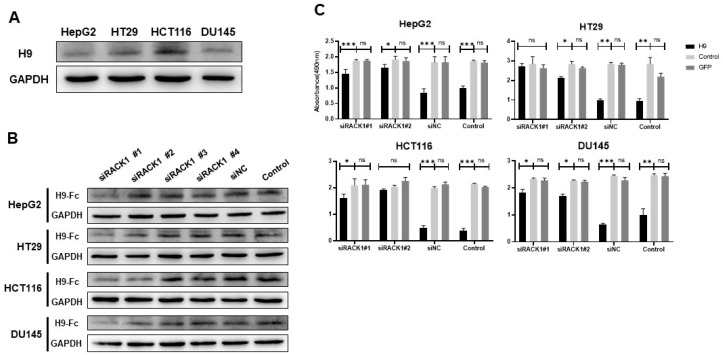
H9 recognizes novel target antigen. (**A**) Western blot analysis demonstrated that the H9 antibody recognizes lysates from HepG2, HT29, HCT116, and DU145 cells. (**B**) Western blot analysis showed that the H9 antibody binds to RACK1 in lysates from siRNA-treated (siRACK1#1–4), siNC-treated, and untreated cells. (**C**) Since siRACK1#1, 2 effectively knocked down RACK1 in tumor cells, the cells treated with this siRNA and the H9 antibody were subsequently analyzed using the MTS assay. Data are presented as mean ± s.e.m. from an experiment independently repeated at least three times. Statistical analysis was conducted using multiple *t*-tests and one-way ANOVA: ns, not significant; * *p* < 0.05, ** *p* < 0.01, *** *p* < 0.001.

The purified H9 antibody was incubated with HepG2, HT29, HCT116, and DU145 cells to identify the target protein specifically bound by the H9 antibody. Immune complexes were isolated by immunoprecipitation. Proteins, which interacted with the H9 antibody, were identified by mass spectrometry. Since the receptor for activated C kinase 1 (RACK1) was dominantly recognized as one of the top matches, it was regarded as the target antigen of the H9 antibody (Figure S2). Western blotting showed that RACK1 was expressed in four tumor cells with the correct molecular weight (Figure 5A). To examine if there was an interaction between the H9 antibody and RACK1 to inhibit cancer cell growth, we used four siRNAs (siRACK1#1–4), a silence control, and a control to knock down RACK1 in HepG2, HT29, HCT116, and DU145 cells (Figure 5B). Since siRACK1 #1 and 2 were successful in diminishing RACK1 levels in these cancer cell lines, we treated the cells with the H9 antibody after the RACK1 silencing. In the absence of RACK1, the H9 antibody treatment did not inhibit cancer cell growth. Conversely, the H9 antibody inhibited cancer cell growth in the control group where RACK1 was not silenced (Figure 5C). These results demonstrate that the H9 antibody suppresses tumor cell growth through its interaction with RACK1.

## 3. Discussion

Our current research showed that the new antibody screening method effectively selects antibodies that cause cell growth arrest and induces apoptosis. Even though the receptor for activated C kinase 1 (RACK1) is well recognized as a potential target protein for various cancers, there is still no commercial therapeutic drug available. Our research suggests that the H9 antibody could be a promising candidate for new cancer treatments.

The landscape of cancer therapy has been significantly transformed over the past few years, with a shift towards more targeted and personalized treatment strategies. Traditional methods like chemotherapy and radiation therapy are being complemented with novel approaches that aim to enhance effectiveness while minimizing side effects. Among these targeted therapies, immunotherapy has emerged as an innovative approach in cancer treatment. It applies the power of the immune system to target and eliminate cancer cells. CAR-T cell therapy, which involves genetically engineering a patient’s T-cells to recognize and attack cancer cells, has achieved significant success, particularly in hematologic malignancies. Despite this, challenges such as toxicity and resistance remain, highlighting the need for continued innovation and refinement [27,28].

The future of cancer therapy is moving towards highly personalized treatments, driven by the genetic and molecular characteristics of individual tumors. Personalized medicine aims to tailor therapies to the specific needs of each patient, maximizing efficacy while minimizing adverse effects. Advances in understanding the molecular mechanisms of cell cycle regulation and immune responses are expected to play a crucial role in this evolution [29,30]. Researchers are also exploring combinations of traditional therapies with novel agents. For example, combining checkpoint inhibitors with established treatments like chemotherapy and radiation is being investigated to enhance therapeutic outcomes and reduce side effects. These combination therapies hold promise for expanding the arsenal of effective cancer treatments [31,32,33].

Cell cycle arrest is a process that inhibits the proliferation of cancer cells by stopping them at specific phases of their growth cycle. Conventional treatments such as chemotherapy and radiation therapy leverage this mechanism to increase the susceptibility of cancer cells to treatment. Recently, photodynamic therapy (PDT) has gained attention for its ability to induce cell cycle arrest. PDT uses photosensitizing agents activated by light to produce reactive oxygen species, which in turn cause cancer cell death through apoptosis, autophagy, and necrosis [34,35]. Moreover, targeting specific checkpoints in the cell cycle with inhibitors like Chk1/2 and ATR has shown potential in sensitizing cancer cells to DNA-damaging agents. These inhibitors can drive cancer cells to bypass cell cycle arrest, leading to cell death via mechanisms like mitotic catastrophe. While some early clinical trials faced setbacks, ongoing research continues to optimize these approaches, offering hope for more effective treatments [36,37].

Combining the antibody screening method with cell cycle base targeting strategies has shown promising synergistic effects. For instance, a selected H9 antibody not only suppresses liver cancer cell proliferation but also inhibits other colon and prostate cancer cells by blocking target protein. Our results indicate that new devised antibody screening methods with any cell function mechanisms will be developed to select a potent functional antibody.

We discovered new antigens for the H9 antibody, particularly the antigen receptor for activated C kinase 1 (RACK1), which is notably important in regulating several cellular mechanisms in cancer. RACK1 is also related to neurovegetative diseases such as amyotrophic lateral sclerosis (ALS) and Huntington’s disease (HD) [38,39].

In 1989, researchers identified RACK1 from a human B-lymphoblastoid cell line. This protein, part of the tryptophan–aspartic acid repeat (WD repeat) protein family, is crucial for various cellular function. It shuttles and anchors proteins, modulates binding protein functions, and engages with nuclear proteins and cell surface receptors. Additionally, RACK1 acts as a scaffold for multiple signaling molecules, influencing processes such as cell growth, migration, development, differentiation, adhesion, and immune response [25]. Notably, RACK1 is upregulated in cancers like colorectal adenocarcinoma, hepatocellular carcinoma, breast cancer, non-small cell lung cancer, and melanoma, highlighting its significant role in cancer progression and development [20,23,40,41,42,43]. RACK1 is markedly upregulated in several cancers like breast, colorectal adenocarcinoma, cervical cancer, hepatocellular carcinoma, and prostate cancer [19,22,25,44,45]. Our study showed that silencing RACK1 reduced the effectiveness of the H9 antibody in these cancer cells, suggesting that targeting RACK1 with H9 could be a promising strategy for cancer treatment. Our finding is consistent with previous reported data. RACK1 overexpression is linked to more aggressive forms of cancers, including prostate, colorectal, and breast cancers, as well as hepatocellular carcinoma, nasopharyngeal carcinoma, and non-small cell lung cancer [19,22,23,25]. Interestingly, RACK1′s role changes in gastric cancer and pancreatic ductal adenocarcinoma, where it appears to act as a tumor suppressor [21,24]. We developed a specific ScFv antibody for RACK1, which has been shown to inhibit cancer growth and migration while inducing G0/G1 phase arrest in cells. These findings highlight RACK1′s potential as a target for antibody-based therapies to combat various solid tumors.

These findings highlight the potential of RACK1 as a crucial therapeutic target in cancer treatment due to its involvement in key cellular processes and its link to cancer aggressiveness. However, the exact mechanisms by which the H9 antibody antagonist against RACK1 triggers cell cycle arrest and apoptosis remain unclear. We are currently examining the characteristics of the RACK1 complex on the surface of solid tumor cells. In our study, the H9 antibody showed growth inhibition across four cancer cell lines, and we plan to extend this investigation to additional colon, liver, and prostate cancer cell lines, as well as breast, lung, and stomach cancer cells. Moreover, ongoing animal studies aim to determine whether the H9 antibody antagonist significantly reduces tumor growth in vivo models.

The use of antibody-based therapies in clinical settings offers several key advantages. Antibodies have high specificity, allowing for the precise targeting of cancer cells while minimizing side effects. This approach also supports personalized therapies tailored to a patient’s genetic makeup or disease profile, enhancing treatment effectiveness. Additionally, antibodies can activate the immune system to attack the targeted cells, creating potential synergistic effects when used alongside other therapies. They can also act as drug carriers, improving targeted drug delivery and reducing overall toxicity.

The development of this novel antibody screening method is particularly promising due to its applicability to all types of solid cancers and its potential to address other challenging diseases. This innovative approach not only deepens our understanding of RACK1′s role in cancer progression but also paves the way for new targeted therapies across a wide range of malignancies and other difficult-to-treat conditions.

## 4. Materials and Methods

### 4.1. Cell Lines and Culture Media

HEK293T, HCT116, HepG2, BEAS-2B, and MCF7 cells were cultured in DMEM medium (Corning, NY, USA) supplemented with a 10% fetal bovine serum (FBS, Gibco, Carlsbad, CA, USA) and a concentration of 1% penicillin/streptomycin (Gibco, Carlsbad, CA, USA). In contrast, HT29, HCT116, AGS, and DU145 cells were maintained in an RPMI 1640 medium (Corning, NY, USA), also enriched with concentrations of 10% FBS and 1% penicillin/streptomycin. The FreeStyle™ 293-F cell line (Invitrogen, Carlsbad, CA, USA) was grown in a FreeStyle 293 expression medium (Invitrogen, Carlsbad, CA, USA). All cell cultures were incubated at 37 °C in a humidified atmosphere containing 5% CO^2^.

### 4.2. Expression and Purification of ScFv-Fc Antibodies

Single-chain Fv (ScFv) genes were derived from a naïve human combinatorial antibody library with a diversity of 1.3 × 10^11^. ScFv-Fc antibodies were generated using the FreeStyle™ 293 expression system (Invitrogen, Carlsbad, CA, USA) with the pFuse-FC vector. The pFuse vector contains the Fc region of human IgG1, spanning from the hinge to the CH3 domain (InvivoGen, San Diego, CA, USA). The ScFv-Fc antibody genes were transiently transfected into FreeStyle™ 293-F cells (Invitrogen, Carlsbad, CA, USA). Antibodies secreted into the culture supernatants were purified using HiTrap Protein A HP columns (GE Healthcare, Chicago, IL, USA) on a BioLogic LP system (Bio-Rad, Hercules, CA, USA). After purification, the buffer was exchanged to PBS solution (pH 7.4) (Corning, NY, USA), and the antibodies were stored at −20 °C.

### 4.3. Lentivirus Transfection

HEK293T cells were seeded at a density of 6 × 10^5^ cells per well in a 6-well plate and incubated overnight. The following day, the cells were transfected using Lipidofect-P (Lipidomia, Republic of Korea) as the transfection agent. The transfection mixture included 1.3 μg of pLV plasmid, 1.3 μg of pCMVD packaging plasmid, and 1.3 μg of pVSVG envelope plasmid. After 4 h of incubation, the culture medium was replaced with 2 mL of fresh growth medium. The cells were then incubated for an additional 72 h. The culture supernatants were collected, and the lentiviruses were aliquoted and stored at −80 °C.

### 4.4. RNA Interference

Small interfering RNA (siRNA) oligonucleotides targeting RACK1, along with a negative control RNAi (siNC), were synthesized by Gene Pharma Co. (Shanghai, China). The siRNAs were transfected into tumor cells using Lipidofect-P (Lipidomia, Seongnam, Republic of Korea) following the manufacturer’s instructions. Forty-eight hours post-transfection, cell lysates were collected for Western blot analysis to assess the effects of the siRNA. Subsequently, the transfected cells were subjected to MTS and colony forming assays. The specific sequences of the siRNAs were as follows Table 1.

### 4.5. Cell Viability Assay

Cells were seeded at a density of 3000 to 6000 cells per well in 96-well plates and subsequently infected with the H9 lentivirus in the presence of 10 µg/mL polybrene transfection reagent (Millipore, Burlington, MA, USA). After 24 h, the culture medium was replaced with 100 µL of fresh growth medium. The cells were then cultured for an additional 4–5 days. Following this, 20 µL of CellTiter 96 AQueous One Solution (Promega, Madison, WI, USA) was added to each well, and the plates were incubated at 37 °C for 3 h. The optical density (OD) was then measured at 490 nm using a SpectraMax 190 Microplate Reader (Molecular Devices, San Jose, CA, USA).

### 4.6. Colony Formation Assay

Cells were seeded at a clonogenic density of 200 to 500 cells per well in 12-well plates. After 24 h, they were treated with the H9 lentivirus and a 10 µg/mL polybrene transfection reagent (Merck Millipore, Darmstadt, Germany) for 12 h. During this treatment period, the medium was changed twice, with a fresh medium added every 3 days. After 10 to 12 days, colonies were fixed with a concentration of 4% paraformaldehyde in PBS for 20 min, washed with PBS, and then stained with a concentration of 0.03% crystal violet in water for 20 min. All experiments were performed in triplicate.

### 4.7. Wound Healing Assay

Cells were seeded at a density of 10^6^ cells per well in 12-well plates and incubated for 24 h. A wound was created by scratching a straight line through the cell monolayer using a pipette tip. A fresh culture medium containing the H9 lentivirus and a 10 µg/mL polybrene transfection reagent (Millipore, Burlington, MA, USA) was then added to the wells. Images of the wound area were captured at 12 h, 24 h, and 48 h intervals. The cell migration rate was assessed by comparing the scratched area at each time point to the initial wound area at 0 h, with the analysis performed using ImageJ software (version 1.53k).

### 4.8. Immunoblotting

Cells were washed twice with ice-cold PBS and lysed using an RIPA lysis buffer (Thermo Scientific, Waltham, MA, USA). The lysates were then centrifuged at 15,000 rpm for 25 min at 4 °C, and protein concentrations were determined using the BCA Protein Assay Kit (Thermo Scientific, Waltham, MA, USA). Proteins were denatured by heating in an SDS-PAGE loading buffer at 70 °C for 5 min, separated by SDS-PAGE, and transferred onto polyvinylidene difluoride (PVDF) or nitrocellulose (NC) membranes using the Trans-Blot Turbo transfer system (Bio-Rad). The membranes were blocked for 1 h with a 5% blocking buffer (a concentration of 0.2% Tween 20 and 5% skim milk powder in TBS), then incubated overnight with primary antibodies against H9, cyclin B1, cyclin D1, cyclin E1, GAPDH, PARP, p21Waf1/Cip1 (Cell Signaling Technology, Danvers, MA, USA), p53 (Millipore, Burlington, MA, USA), or Bax (Cell Signaling Technology, Danvers, MA, USA). Following primary antibody incubation, the membranes were washed three times with TBST (Tris-buffered saline with 0.1%Tween 20) for 15 min each. They were then incubated with HRP-conjugated secondary antibodies (anti-IgG1, anti-rabbit, or anti-mouse; Cell Signaling Technology, Danvers, MA, USA) for 1 h. The membranes were washed again three times with TBST for 10 min each and developed using Clarity Western ECL Substrate (Bio-Rad) and SuperSignal™ West Dura Substrate (Thermo Scientific, Waltham, MA, USA).

### 4.9. Flow Cytometry

Cell cycle analysis was conducted using propidium iodide (PI) staining. Prior to analysis, cells were treated with the H9 lentivirus and a 10 µg/mL polybrene transfection reagent (Millipore, Burlington, MA, USA). The harvested cells were fixed in a concentration of 4% paraformaldehyde in PBS at 4 °C for 1–2 h, then incubated with a solution containing 1 µg/mL PI (Invitrogen, Carlsbad, CA, USA), 0.1% Triton X-100, and 0.5 µg/mL RNase A. Cell cycle phases were analyzed using an LSRFortessa X-20 flow cytometer (BD Biosciences) and FlowJo X software (version 10.0.7r2, BD Biosciences, San Jose, CA, USA). For apoptosis analysis, cells were treated with the H9 lentivirus and a 10 µg/mL polybrene transfection reagent. After harvesting, the cells were washed twice with ice-cold PBS and resuspended in binding buffer (10 mM Hepes, 0.14 M NaCl, 2.5 mM CaCl2 in distilled water). They were then incubated with 1 µg/mL PI and 20 µL annexin V-Pacific Blue (Invitrogen, Carlsbad, CA, USA) for 20 min on ice. Apoptosis was evaluated using the LSRFortessa X-20 flow cytometer and analyzed with FlowJo X software.

### 4.10. Immunoprecipitation and Liquid Chromatography–Mass Spectrometry

Cells were lysed using an IP lysis/wash buffer (Thermo Scientific, Waltham, MA, USA). The lysates were then incubated overnight at 4 °C with an H9 antibody and 20 μL of Pierce Protein A/G Plus Agarose (Thermo Scientific, Waltham, MA, USA). The eluted proteins were subsequently analyzed by liquid chromatography–mass spectrometry (LC-MS) at the Korea Basic Science Institute (Ochang, Republic of Korea).

## Figures and Tables

**Table 1:** The specific sequences of the siRNAs.

Name	Sequence
RACK1#1	5′ CCAUCAAGCUAUGGAAUACTT 3′
RACK1#2	5′ GCUAUGGAAUACCCUGGGUTT 3′
RACK1#3	5′ CCUUUACACGCUAGAUGGUTT 3′
RACK1#4	5′ GACAUCAUCAUGUGGAAGCTT 3′
siNC	5′ GACCAUCAUCAUGUGGAAGCTT 3′

## Data Availability

Data is contained within the article or Supplementary Materials.

## Supplementary Materials

The following supporting information can be downloaded at: https://www.mdpi.com/article/10.3390/ph17101303/s1, Figure S1: Antibody Selection for G0/G1 Phase Cells Using Flow Cytometry; Figure S2: Target identification by mass spectrometry.
