# Supplementary material for: An Antagonist Antibody That Inhibits Cancer Cell Growth In Vitro through RACK1
Source: Pharmaceuticals (Basel). 2024 Sep 30;17(10):1303. doi: 10.3390/ph17101303 (PMC11510629; doi:10.3390/ph17101303)
Supplement: Supplementary file 1 [file pharmaceuticals-17-01303-s001.zip › pharmaceuticals-3200534-supplementary.pdf]

An Antagonist Antibody That Inhibits Cancer Cell Growth In Vitro through RACK1

Ji Hoe Kim †, Eun Ji Lee † and Kyung Ho Han *

Department of Biological Sciences and Biotechnology, Hannam University, Daejeon 34054, Republic of Korea
* Correspondence: kyungho1@hnu.kr; Tel.: +82-42-629-8770
† These authors contributed equally to this work.

Supplementary information

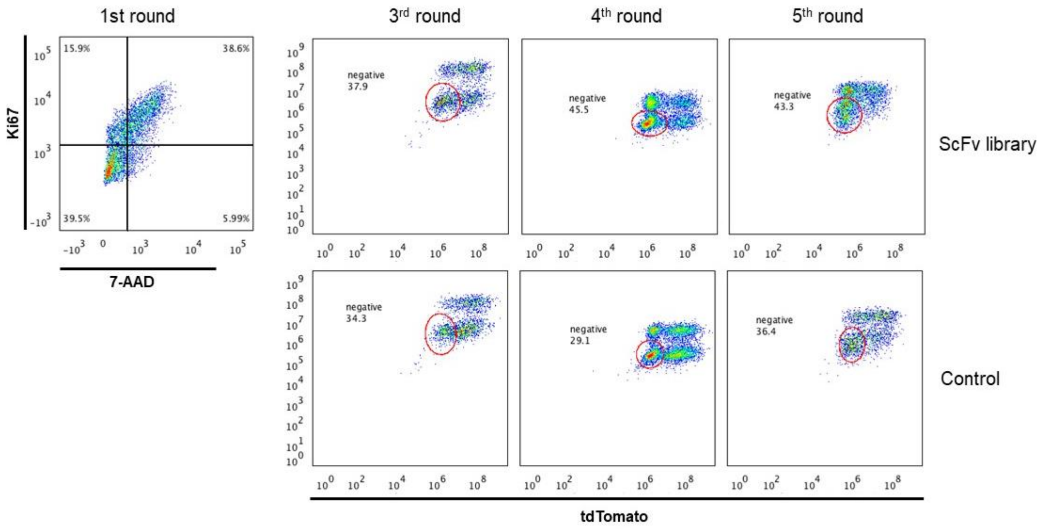

Figure. S1. Antibody Selection for G0/G1 Phase Cells Using Flow Cytometry

After three days of treatment, the cells were sorted using flow cytometry. During the first round, cells were sorted based on the Ki67-negative population. Genomic DNA was then extracted and used to clone a focused lentiviral library. In the subsequent 3-5 rounds, cells were sorted based on the BrdU-negative population, indicating they were in the G0/G1 phase of the cell cycle. Each round produced a more focused antibody library specific to the G0/G1 phase in the HT29 cell line compared to previous rounds. With each round, there was an increased population of cells in the G0/G1 phase compared to the control.

A

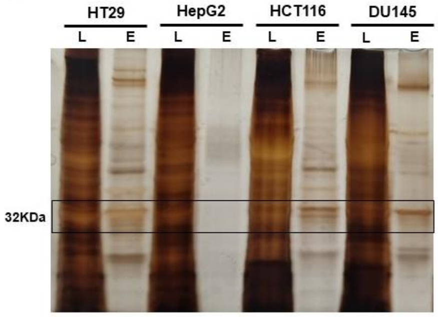

B

Hit name	Hit #
Receptor of activated protein C kinase 1	8
ELAV-like protein 1	6
Triosephosphate isomerase	5
Phosphoglycerate kinase 1	2

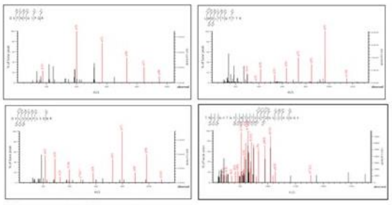

>P63244, Receptor of activated protein C kinase 1 OS=[Homo sapiens]

```
  1 MTEQMTLRGT LKGHNGWVTQ IATTPQFPDM ILSASRDKTI IMWKLTRDET
 51 NYGIPQRALR GHSHFVSDVV ISSDGQFALS GSWDGTLRLW DLTTGTTTRR
101 FVGHTKDVLS VAFSSDNRQI VSGSRDKTIK LWNTLGVCKY TVQDESHSEW
151 VSCVRFSPNS SNPIIVSCGW DKLVKVWNLA NCKLKTNHIG HTGYLNTVTV
201 SPDGSLCASG GKDGQAMLWD LNEGKHLYTL DGGDIINALC FSPNRYWLCA
251 ATGPSIKIWD LEGKIIVDEL KQEVISTSSK AEPPQCTSLA WSADGQTLFA
301 GYTDNLVRVW QVTIGTR
```

Figure. S2. Target identification by mass spectrometry

(A) Cell lysates of four tumor cells were incubated with H9 antibody for immunoprecipitation. Immuno-precipitated elutes were separated on SDS/PAGE gels that were silver-stained. (B) LC-MS analysis identified several candidates as antigen. RACK1 peptides are highlighted in blue.
